# Roles of Hormone Replacement Therapy and Menopause on Osteoarthritis and Cardiovascular Disease Outcomes: A Narrative Review

**DOI:** 10.3389/fresc.2022.825147

**Published:** 2022-03-28

**Authors:** Yixue Mei, Jennifer S. Williams, Erin K. Webb, Alison K. Shea, Maureen J. MacDonald, Baraa K. Al-Khazraji

**Affiliations:** ^1^Department of Kinesiology, Faculty of Science, McMaster University, Hamilton, ON, Canada; ^2^Department of Obstetrics and Gynaecology, Faculty of Medicine, McMaster University, Hamilton, ON, Canada

**Keywords:** menopause, osteoarthritis, cardiovascular disease, hormone therapy, aging/ageing, cardiovascular disease risk factors, woman/women

## Abstract

Osteoarthritis (OA) is a highly prevalent condition characterized by degradation of the joints. OA and cardiovascular disease (CVD) are leading contributors to disease burden worldwide, with a high level of overlap between the risk factors and occurrence of both conditions. Chief among the risk factors that contribute to OA and CVD are sex and age, which are both independent and interacting traits. Specifically, the prevalence of both conditions is higher in older women, which may be mediated by the occurrence of menopause. Menopause represents a significant transition in a women's life, and the rapid decline in circulating sex hormones, estrogen and progesterone, leads to complex physiological changes. Declines in hormone levels may partially explain the increase in prevalence of OA and CVD in post-menopausal women. In theory, the use of hormone therapy (HT) may buffer adverse effects of menopause; however, it is unclear whether HT offers protective effects for the onset or progression of these diseases. Studies have shown mixed results when describing the influence of HT on disease risk among post-menopausal women, which warrants further exploration. The roles that increasing age, female sex, HT, and CVD play in OA risk demonstrate that OA is a multifaceted condition. This review provides a timely consolidation of current literature and suggests aims for future research directions to bridge gaps in the understanding of how OA, CVD, and HT interact in post-menopausal women.

## Introduction

Osteoarthritis (OA) is a progressive musculoskeletal disease involving the breakdown of cartilage and bone, and is one of the leading causes of pain and disability worldwide (1). An estimated 654 million individuals globally have OA, with higher prevalence in women (~22%) than in men (~12%), and sex-differences becoming apparent only after age 50 (2). Recent literature has found that up to 60% of individuals with OA had additional chronic conditions (3). Specifically, numerous studies have reported that individuals with OA have a higher risk for cardiovascular disease (CVD) (4–6), a disease which leads to over 17.3 million deaths per year worldwide (7). Collectively OA and CVD create a considerable healthcare burden and share complex risk factors that contribute to a large fraction of disability, and to a greater extent in the older population. Post-menopausal women are especially vulnerable to the onset of both OA and CVD, which may suggest that the occurrence of menopause and the associated physiological changes play a mediating role in the increased co-existence of OA and CVD.

Menopause represents a significant transition in a woman's life and is characterized by the cessation of the menstrual cycle and the subsequent dramatic decrease in levels of sex hormones estrogen and progesterone. Though testosterone also decreases in post-menopausal women, this decrease is more gradual, and the effects of androgens on women's health following the menopause transition is not fully understood (8, 9). The roles of testosterone and testosterone replacement therapy on OA and CVD risk in post-menopausal women fall outside the scope of this narrative review.

The relationship between OA and menopause was first described by Drs. Cecil and Harper in 1925 (10), and by 1952 was termed “menopausal arthritis” (11). Since then, several research studies have investigated the influence of the changing sex hormone profile during the menopausal transition on OA onset and progression. The higher prevalence of OA in older women is, in part, a result of estrogen deficiency (12), making hormone replacement therapy (HT) an important avenue to explore with consideration to preservation of joint health in OA (13, 14). Additionally, the apparent role of estrogen in maintaining cardiovascular health may influence CVD risk in older women with estrogen deficiency; however, prior research studies have presented mixed findings (15). The link between OA and CVD in the context of menopause is unclear, and the impact of HT for women with these conditions requires further consolidation of current literature. In addition, the relationships between OA and CVD and their associated risk factors may be facilitated by both biological and societal determinants of health. Specifically, factors such as gender identity (16, 17) and social disadvantage (18) are influencers of disease risk and health outcomes, but are often overlooked in biomedical research.

The purpose of this review is to synthesize existing literature examining the influence of menopause and HT on OA and CVD in women from a biomedical perspective, while applying an intersectional lens that considers socially-constructed gender identity and expression, age, ethnicity and socioeconomic factors. Specifically, biomedical literature pertaining to OA, CVD, menopause, and HT use within the recent decades were reviewed. Further searches were conducted to examine socioeconomic factors on OA and CVD, and how they interacted with menopause and HT primarily from a risk and prevalence perspective. It is important to note that this is a broad topic, thus the scope of the paper has been limited to certain factors of influence, though the authors acknowledge the vast spanning of this research field.

Given that women are disproportionately impacted by both OA and CVD and have been historically under-studied in biomedical and clinical research fields (19), this review provides a timely overview of current literature and highlights future research directions required to address knowledge gaps in the role of HT on OA, and subsequent CVD comorbidities, in women following menopause. Specifically, this review will cover literature on CVD and HT as important considerations when implementing interventions for OA management.

## OA and Menopause

Osteoarthritis (OA) is one of the leading causes of chronic pain and disability, and is primarily characterized by excessive cartilage degradation, abnormal subchondral bone growth and sclerosis, and synovial inflammation (13). An abundance of evidence highlights the significant influence OA has on accelerating the onset of functional limitations and disability (20). The decline in physical activity in those living with OA subsequently contributes to both physical limitations (21) and diminished mental health (22, 23). The most prevalent sites of OA, namely with increasing age, are in the hand, knee, and hip (24, 25). It is important to acknowledge that these different locations of OA may potentially have different risk associations with menopause due to their differing etiology (24). Specifically, OA of the knee or hip, or weight-bearing OA, has greater incidence in post-menopausal women (24) and is more greatly associated with CVD risk than hand OA (26, 27). This review distinguishes between the subgroups of OA where possible.

Incidence and prevalence of OA are significantly greater in women than in men after the age of 50 (28–30), and there is a greater difference in the loss of cartilage volume between the sexes that becomes more prominent with age (31). Particularly in knee and hip OA, the prevalence and risk rapidly increase from menopausal age (around 50 years) to 75 years when compared to men of similar age (32). One large retrospective study conducted in Spain of over 5 million people reported that in adults who were 50 to 75 years of age, a diagnosis of hip and/or knee OA was 1.5–2 times greater in women than men (27). The increased OA prevalence and cartilage decline in post-menopausal women suggests that there could be a role of sex hormones on maintenance of joint health (33). Several earlier reviews investigating the effect(s) of endogenous sex hormones in post-menopausal women on OA concluded that there is an association between decreased endogenous estrogen levels and an increased risk and incidence of OA (12, 29, 30, 34). More recently, a study found that lower serum estradiol and progesterone were associated with increased knee effusion-synovitis in post-menopausal women, whereas higher levels of endogenous progesterone were associated with greater cartilage volume (35).

### Animal Studies

Animal studies provide a unique lens to study OA as these studies can isolate and manipulate individual variables (e.g., knock-out of one specific gene, influence of one specific hormone) and assess outcomes, which is challenging to accomplish even in controlled human trials. Ovaries are commonly removed in animal models to simulate post-menopausal conditions. A systematic review identified 16 high-quality animal studies (rodents, pigs, sheep, monkeys) that utilized the ovariectomy (OVX) technique to study the effects of reduced sex hormones on the development of OA (36). Of the 16 studies examined, 11 showed a detrimental effect of OVX on the outcome measures for OA (cartilage damage/degradation and mechanical defects), 4 showed no effect, and 1 showed a positive effect (36). Notably, several of these studies found that CTX-II (a biomarker of collagen type II breakdown) was elevated in mice that underwent OVX compared to the control animals, consistent with data previously reported in post-menopausal women (37, 38). Recent animal studies have provided mechanistic insight into how sex hormones may mediate the progression of OA in animal models. One study determined that OVX induced more severe OA in AMP-activated protein kinase (AMPK) knock-out mice, suggesting that estrogen may be acting through AMPK signaling cascades to protect articular cartilage (39).

### Human Studies

Estrogen and progesterone receptors are present on many bone and cartilage tissues (40), where sex hormones bind and elicit downstream signaling effects. Several excellent reviews detail the role that estrogen and, to a lesser degree, progesterone, play in regulating joint and bone health (13, 40, 41). Briefly, estrogen increases proteoglycan production (42), decreases inflammation and reactive oxygen species (43, 44), and regulates calcium signaling in chondrocytes (13, 45). Estrogen also regulates subchondral bone turnover. It has been shown that OVX monkeys experience higher indices of bone turnover in subchondral bone than cancellous bone in comparison to the controls (46). In fact, OA is worsened in OVX-treated rabbits following methyl-prednisone-induced osteoporosis compared to controls, indicating that estrogen might have a dual effect on OA: a more direct effect on cartilage, and an indirect effect on subchondral bone (13). In comparison, the role of progesterone is not well understood; however, it may contribute to the maintenance of cartilage volume through suppressing production of matrix metalloproteinases (47). The role of sex hormones in moderating joint health in the context of OA risk requires future studies to isolate for the mechanistic effects and warrants further research in both animal and human populations.

Social disadvantage determinants, sometimes studied through racial differences, socioeconomic determinants, or perceived pain, have been proposed to influence OA outcomes (48, 49). Ethnicity has been suggested to further augment OA risk and disability (50, 51). Specifically in post-menopausal women, African-American, Hispanic, or Aboriginal ancestry are most susceptible to OA (50). Early research investigating factors such as education, income, and occupation have found that lower socioeconomic statuses and nonprofessional occupations were more strongly associated with arthritis outcomes, even after controlling for individual factors such as female sex and age (48). The severity of pain experienced in OA is associated with the degree of bone attrition and effusion-synovitis, both of which appear to be influenced by sex hormones (52). The role of estrogen in pain modulation is complex, as it may attenuate acute inflammatory pain but it has both anti- and pro-nociceptive effects (53). A foundational study reported that while women with OA experience significantly higher levels of pain and disability than men with OA, after adjusting for catastrophizing tendencies, or the tendency to exaggerate painful stimuli and take on a helpless role, the gender differences were eliminated (16). Recent work has shown that lower levels of emotional expression were associated with more pain catastrophizing in people with OA. However, when effective communication of pain is high, the act of pain catastrophizing becomes weaker, which may suggest that communication of pain is influenced by sociocultural factors, alongside the underlying biological sex factors (54). We recognize that social determinants of health are complex, multi-tiered with many aspects, and are not specifically or solely associated with OA. However, when considering post-menopausal women who may be at an increased risk of experiencing pain associated with OA due to attenuated sex hormones or general social disadvantage, better understanding of non-biomedical aspects of disease could have the potential to improve treatment and care strategies that women receive, and thereby further improve prognosis.

## CVD and Menopause

Men have a higher prevalence of CVD in comparison to women until the menopause transition (7). According to the INTERHEART global case-control study conducted across 52 countries, women experience their first acute myocardial infarction ~9 years later than men (55). The INTERHEART study identified that there are nine modifiable risk factors associated with CVD, some of which are differentially associated with risk between sexes (55). Further, this study found that sex differences in acute myocardial infarction were largely (80%) explained by these modifiable risk factors in men compared to women (55). The risk factors may result in increased risks for conditions such as atherosclerosis (56–59) or metabolic syndrome (55, 59–61), which directly contribute CVD onset. However, sex differences in CVD prevalence cannot be examined simply through a lens of biological sex as disease progression is influenced and elevated by intersections of (but not limited to) age, sex, gender, ethnic background, socioeconomic status, and access to care (17, 18).

Recent research by Pelletier and colleagues (17) done in a cohort of middle-aged adults (mean age of 48 years) reported that higher gender scores associated with feminine gender, as opposed to female sex, are associated with risk for CVD, indicating that socially constructed gender roles and their expression play a mediating role in CVD onset. Furthermore, Anand and colleagues (18) examined the role of social disadvantage (as an index of social and economic factors) and ethnicity (i.e., South Asian, Chinese, Aboriginal, and European ancestry) on CVD risk across middle-age (mean age of 50 years). The authors identified social disadvantage as an independent predictor of CVD, independent of age, sex, and ethnicity. Further, this study reported that women, in particular older women across all ethnic groups, had a higher proportion of social disadvantages than men, where ethnicity further contributed to the unequal risk of CVD. Specifically, compared to European participants, Aboriginal and South Asian participants were at a significantly higher risk (trend for South Asian) of CVD, while Chinese participants were at a lower risk of CVD, even after social disadvantage was accounted for in the analysis (18). Taken together, it is important to consider both biomedical and sociocultural risk factors when examining sex differences in CVD risk; however, minimal research has been conducted examining the role of non-biomedical factors like gender and ethnicity in CVD risk across the menopause transition, thus warranting further research.

Changes associated with the menopause transition drastically increase the risk of CVD among women as compared to men, such as the loss of estrogen receptors in post-menopausal women (62), years since menopause, and comorbid diseases (63). Furthermore, CVD risk is increased during peri- and post-menopause due to changes in lipids (i.e., cholesterol, triglycerides, lipoprotein A), body composition, and vascular function, among other factors underlying CVD progression (64). A recent publication from the American Heart Association identified early age of menopause (i.e., <45 years) as a risk factor for various CVDs (64), even when adjusting for age, ethnicity, and other traditional risk factors (57). Surgical menopause (i.e., the removal of both ovaries) may further increase the risk of CVD, particularly if the surgery occurred before the age of 40 years (e.g., <35–39 years) and follow-up care did not include HT to replace the loss of endogenous hormones (65).

Data from both longitudinal and cross-sectional studies support the theory that the menopause transition, and specifically the reduction in endogenous estradiol, underlies the age-related sex differences observed in the prevalence of CVD (66). Estradiol plays a cardioprotective role in improving vascular function as highlighted by studies using cell, animal and human models (15, 67). Estradiol may exert protective influence on the vasculature through the estrogen receptor – endothelial nitric oxide synthase pathway, increasing the production of the potent vasodilator nitric oxide (68). Further mechanisms of action include the ability for estradiol to reduce oxidative stress and increase antioxidant protection, increase vascular cell survival, reduce arterial fibrosis development, and protect from vascular injury, as detailed extensively in previous reviews (15, 67).

Recent research by Iwamoto and colleagues (69) examining internal carotid artery shear-mediated dilation as an early predictor of CVD found that dilation was impaired in peri- and post-menopausal women, as compared to pre-menopausal women. Importantly, women included in this trial were not using any type of medication, although there was no exclusion of prior use of HT. Furthermore, this study reported that the carotid artery dilation was improved with higher serum estradiol levels, even after controlling for age. These results point to an intrinsic role of estrogen in the regulation of CVD risk, independent of aging, through the menopause transition (69). Additionally, investigations into subclinical markers of CVD progression indicative of vascular disturbance also suggest dysfunction emerges across the menopause transition. Cross-sectionally, brachial artery flow mediated dilation was found to be progressively lower in late peri-menopause and post-menopause compared to early peri-menopause and pre-menopause (70). Similarly, in studies that have performed repeated testing, the menopause transition was associated with preclinical indices of vascular dysfunction including increased carotid intima-media thickness and increased aortic pulse wave velocity at follow-up (71, 72). Overall, despite some conflicting evidence, estradiol may be mechanistically linked to the risk of CVD, particularly across the menopause transition.

## OA, CVD, and Menopause

**Figure 1:**
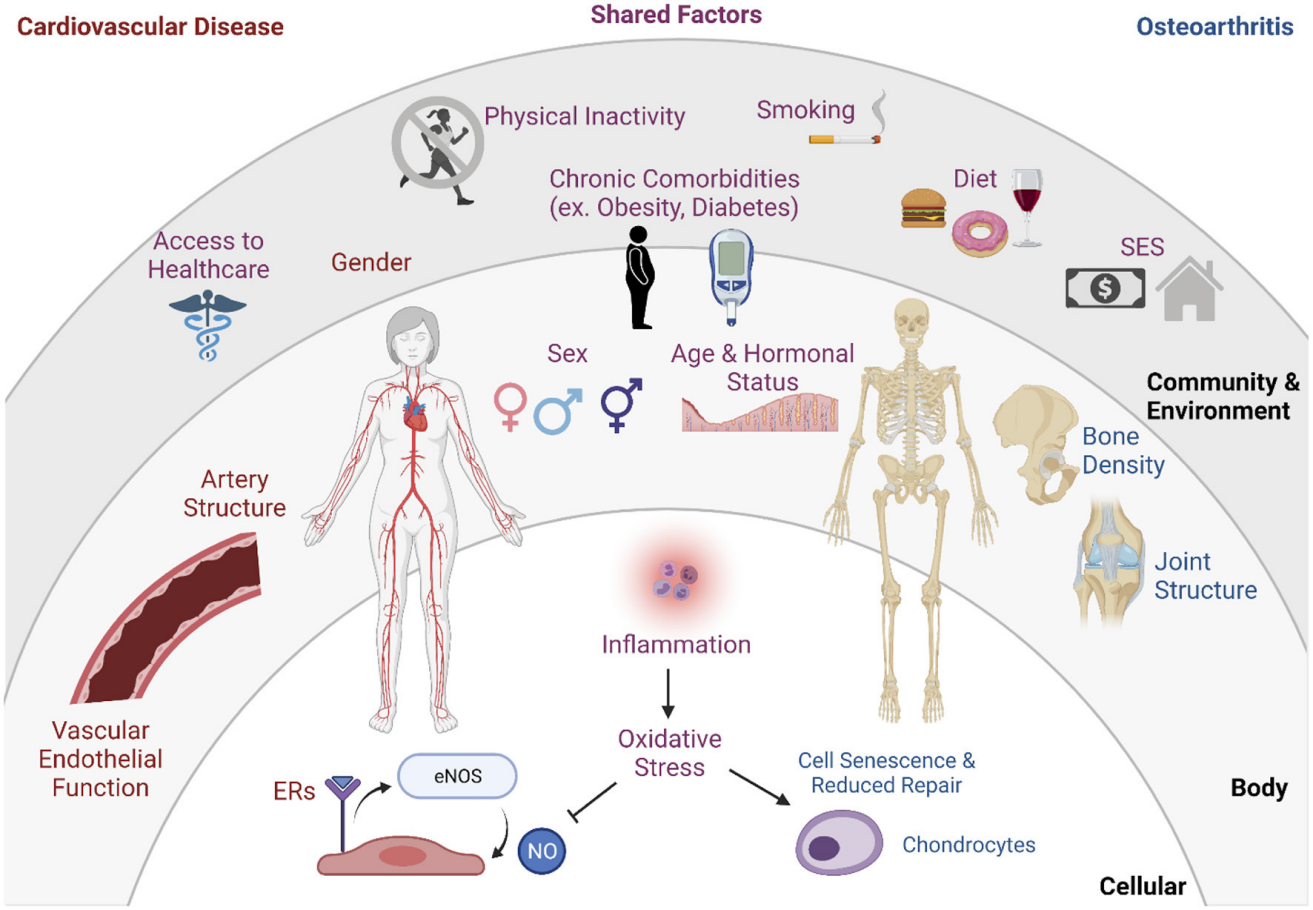
Interaction of risk factors of OA and CVD. Factors with research to support connection to OA alone: cell senescence & reduced repair, chondrocytes, bone density, joint structure. Factors with research to support connection to CVD alone: gender, artery structure, vascular endothelial function estrogen receptors (ERs), eNOS (endothelial nitric oxide synthase), nitric oxide (NO). Shared risk factors include access to healthcare, physical inactivity, chronic comorbidities, smoking, diet, socioeconomic status (SES), sex, age, hormonal status, inflammation, oxidative stress.

The interrelationship of OA and CVD can be partially explained through shared risk factors, overlapping etiology, and various indirect causes (Figure 1) (6). This section aims to highlight the possible mechanisms that link the OA and CVD pathologies and concludes by proposing several ways menopause plays a role in the comorbidities.

Several traditional CVD risk factors, including age, sex, obesity, cholesterol levels, and smoking, all elevate the risk of OA development and progression (5, 92). Aging affects the health of the heart and vasculature, and is associated with increased thickening and stiffening of vessels, which results in hypertension and heightens the risk of developing vascular-related conditions (92). As discussed previously, circulating sex hormone levels influence the levels of adiposity within the body, which in turn influences OA and CVD disease risk and etiology (92). For example, conditions such as diabetes and obesity are risk factors and precursors to the development of both OA and CVD, through inflammation and endothelial dysfunction mechanisms (92). Lifestyle habits, such as smoking and physical inactivity due to pain during movement, are also common risk factors for OA and CVD, and also act through altering endothelial health which contributes to atherosclerosis (93). Finally, another proposed underlying mechanism is the acute or chronic inflammation that is commonly associated with OA, which plays a role in the progressive degradation of joint health. This inflammation also affects the vasculature that supplies bone, and may play a role in increasing CVD-related incidents through the development of atherosclerosis (5, 92). Collectively, these shared risk factors and overlapping etiology point toward a potential disease phenotype that may result from these highly comorbid conditions, where various interactions and synergistic effects influence the severity of the conditions.

Disease management methods may, in part, contribute to the observed comorbidities. Pharmacological methods of managing OA may increase the risk of CVD. Namely, nonsteroidal anti-inflammatory drugs (NSAIDs) are frequently prescribed to relieve joint-related pain in OA patients and have been related to increased precursors of CVD (6, 92). NSAIDs act through inhibiting the production of prostaglandins; however, the interference of prostaglandin production results in adverse effects on vascular health, including altered kidney function resulting in increased blood pressure that subsequently increases the risk for heart failure (94, 95). Additionally, in the case of severe OA where surgical intervention is required, the event of surgery has been proposed to be a mediator between OA and CVD (96). One study found that hand, knee, and hip joint replacement increased the risk of venous thromboembolism, and subsequently the occurrence of cardiac events (96).

In post-menopausal women, the relationship between OA and CVD is further exacerbated through the decrease in circulating estrogen with menopause. A higher OA prevalence in women over 50 has been, in part, attributed to the absence of musculoskeletal-protective effects of estrogen (92). In addition to overlapping biomedical and physiological factors, it is important to consider the effects of environmental and societal contributors to disease. Very few studies have examined the intersection of OA and CVD through lenses of socioeconomic status, food accessibility, and access to care (Figure 1), but it is important to acknowledge these effects on disease onset, progression, and management (18).

## The Role of Hormone Therapy (HT) in Menopause

Estrogen therapy is typically given either with progesterone, or alone (for women without a uterus), to mimic ovarian hormones and alleviate bothersome symptoms of menopause, particularly vasomotor symptoms. When prescribing HT, several factors are considered, including the patients' pre-existing medical conditions, contraindications (i.e., active thromboembolic disease). The risk for development and/or progression of certain diseases (i.e., CVD) may increase based on prior health factors, as well as with the type of HT used (i.e., oral versus transdermal estrogens) (79, 86). The following sections will detail the influence of HT independently on OA and CVD risk, and then discuss the interaction of the comorbidities with HT.

## The Influence of HT on OA Risk

Since the 1980s, several studies have sought to determine the effects of HT on OA incidence, prevalence, and progression. As previous sections have highlighted, there is a strong rationale for utilizing exogenous sex hormones to mitigate the risk of disease: (1) greater OA prevalence in post-menopausal women, (2) the negative association between cartilage damage and circulating levels of sex hormones, and (3) the presence of estrogen and progesterone receptors on joint tissues.

### Animal Studies

Research on animals (rodents, sheep, miniature pigs) have highlighted that estradiol supplementation can inhibit changes in collagen turnover induced by OVX (36). Differences in the OA phenotype throughout the progression of the disease may influence the effect of ET on bone and cartilage function. Specifically, the early stages of OA are characterized by increased subchondral bone turnover and bone loss, whereas in the late stages of OA there is reduced subchondral bone turnover and sclerosis (97). It is possible that ET may have beneficial effects in early OA but may have limited or even harmful effects when treating late OA. The age when beginning HT (i.e., the timing) may be an important consideration in OA (98, 99), like the “timing hypothesis” prevalent in HT usage in CVD (64) (discussed in the following section).

### Human Studies

The first longitudinal study to examine the effects of HT on OA diagnosis followed 551 older women, (aged 63–91) over a 10-year period and found that the current use of estrogen therapy (ET) had a moderate, but not statistically significant, protective effect against the worsening of radiographic knee OA (100). An additional and more recent study in 17,200 older women (3,440 women with OA, 13,760 women without OA, mean age ~51 years) found that menopause was a risk factor for OA, and women who initiated HT shortly after menopause were at reduced risk of OA, with the protective effects disappearing after HT cessation (98). In contrast, a case-control study comparing women with OA to age-matched controls found that current HT use was not associated with OA risk whereas past HT use modestly decreased the risk of developing OA (101). Both studies classified women as either never, current or past users of HT, but the type of HT or duration of treatment were not considered (100, 101). Therefore, based on these early incidence studies, it remains unclear if ET offers a protective effect on OA diagnosis and progression. The Women's Health Initiative conducted a blinded randomized control trial that included over 26, 000 women aged 50–79 assigned to receive either estrogen and progesterone, estrogen-only, or placebo in standardized dosages (conjugated equine estrogen 0.625 mg and medroxyprovera acetate (such as Depo-Provera) 2.5 mg) over an average duration of 7 years (102). The Women's Health Initiative found that the women receiving the estrogen-only treatment had significantly lower rates of overall arthroplasty (surgical management of severe OA), but when stratified by joint of interest, only hip arthroplasty remained slightly lower in the ET group; the occurrence of knee arthroplasty was not significantly different among the groups (103). In the estrogen and progesterone group, there was no association between total, knee, or hip arthroplasties with treatment (103). Similarly, in a separate randomized control trial, estrogen and progesterone HT over 4 years did not mitigate knee pain or degree of disability, measured by the WOMAC OA index, in 969 older women (mean age of 66 years) with CVD (compared to controls) (82). Together, these studies support a potential protective effect of ET on OA incidence which may be attenuated when progesterone is also administered; however, additional work should be completed to explore this relationship.

A systematic review conducted by Tanamas et al. in pre- and post-menopausal women suggested that ET may help with maintaining healthy cartilage and bone turnover, as measured by serum and urinary biochemical markers of bone and cartilage breakdown (CTX-I and CTX-II) (12). A different review conducted by Karsdal et al. summarized structural benefits of estrogens and protective effects for joint inflammation and degradation, suggesting associations between the roles of estrogen and the etiology of OA (104).

Several limitations are important to consider when interpreting the literature on HT and OA, such as duration of HT use, age of HT administration and cessation, type, and dosage of HT as well as general adherence to treatment. Aside from randomized control trials, many cross-sectional or longitudinal studies group HT usage into categories such as “current use,” “past use” or “never use” without taking into consideration additional information on HT. There appears to be differences in OA incidence in response to different types of HT (i.e., estrogen alone versus estrogen and progesterone) which is also not captured through this type of analysis. Not accounting for the details of HT usage introduces a considerable amount of variability. For example, women who utilize HT in their 70's are much more likely to present with significant cartilage damage than women in their 40's simply due to the “wear and tear” nature of the disease (101). Anecdotally, general musculoskeletal symptoms during menopause appear to improve with HT when used early in the menopausal transition (98, 99), but this has not been analyzed in randomized control trials (99).

## The Influence of HT on CVD Risk

Over the last two decades, the potential role of HT for the prevention of CVD has been thoroughly investigated. Several factors have been found as important determinants of the outcomes, such as age since menopause (i.e., “the timing hypothesis”), the composition of HT (e.g., estradiol alone, estradiol with progesterone/progestins), and the route of administration, which have been well detailed in a recent review (64). Longitudinal trials such as the Heart and Estrogen/Progestin Replacement Study (105) and the Women's Health Initiative (106) in postmenopausal women (mean age of ~67 and ~63 years, respectively) were conducted to examine the effects of HT on risk for disease and all-round mortality, but were not found to improve the CVD risk outcomes. However, from these trials, the age of the participants and time since menopause, along with the existing presence of CVD, were discovered to be important factors to consider when examining the potential benefits associated with HT.

Specifically, it appears that the beneficial impacts of HT on mitigating CVD risk are apparent within 10 years of menopause onset, and generally in those younger than 60 years old; this phenomenon has been termed the “timing effect” of HT (107). For example, recent meta-analyses conducted have reported a decrease in CVD risk and mortality with HT in early post-menopausal women (i.e., within 10 years of menopause onset), though there was an increased risk for venous thromboembolism compared to placebo or no treatment conditions (108, 109). In contrast, in late post-menopausal women (i.e., >10 years since menopause onset), there was no change in CVD risk and mortality with HT, though there was an increased risk of stroke and venous thromboembolism (108, 109). Further, the latter meta-analysis observed a ~25% reduction in mortality in younger post-menopausal women (i.e., <60 years old; average: 55 years) using HT compared to no treatment after an average of 5 years follow-up, providing further support to the notion that HT may benefit early post-menopausal women (109). However, this meta-analysis combined trials of women using oral and transdermal estradiol or conjugated equine estrogen, with or without the presence of progesterone or a progestin (e.g., medroxyprogesterone acetate), making it difficult to draw conclusions regarding the type and dose of HT and its impacts on mortality. More recent research such as the Early vs. Late Intervention Trial with Estradiol, reported reduced age-induced increases in carotid intima-media thickness, an early stage of atherosclerotic progression, with early post-menopausal women (i.e., <6 years after menopause) after ~5 years of oral 17β estradiol [(1 mg/day) with progesterone (45 mg vaginal gel) for 10 days in a 30-day cycle] in comparison to late post-menopausal women (i.e., more than 10 years after menopause) (110).

## Current clinical Recommendations

HT is a common treatment in helping manage bothersome vasomotor symptoms (e.g., night sweats, hot flashes) in post-menopausal women, with a large variation in the type, method of administration, duration of administration, pre-existing health risk factors, and time since menopause that all collectively affect the efficacy and disease risk (86, 87, 111) (Table 1). Current guidelines from the International Menopause Society, the North American Menopause Society, and the European Menopause and Andropause Society collectively state that HT remains the most effective treatment for menopausal vasomotor symptoms, with data to support greater benefits in earlier initiation of HT following menopause (~50–59 years of age) (112–115). This section serves to highlight key studies and reviews in this field examining OA and CVD health outcomes using different forms of HT. A newer option for HT is a selective estrogen receptor modulator (SERM), which has both estrogen receptor agonist and antagonist properties that are tissue dependent (85). SERMs have long been used for the treatment of osteoporosis but are infrequently considered for OA, with no trials published that have examined the effects (14, 104). Research investigating the effect of SERMs on CVD has been mixed (90, 91), and further research is needed. Finally, it is important to note that although this review focused on different types of hormonal treatments, there are also non-hormonal therapies used to improve menopausal symptoms which fall outside the scope of this review (87).

**Table 1:** Overview of the influence of HT on OA/CVD outcomes.

**Type of HT**	**Mechanism of HT**	**OA risk/outcome**	**CVD risk/outcome**
Estrogen^*^	Estrogen receptors in joint tissues (66)	No clear association (73)	Potentially beneficial if started early, increases clearance of LDL and synthesis of HDL (74) Indicated cardiovascular benefits in women with hysterectomy (75)
Oral CEE	Mixture of estrogen compounds, antagonistically bind to estrogen receptors (76)	Modest reduction in joint pain, lower rates of arthroplasty (14, 77)	Decreased formation of oxidized LDL and HDL (78)
Oral Progesterone^**^	Stabilizes endometrial lining (79)	Aids in bone remodeling (80)	May provide short-term cardiovascular safety (81)
Estrogen-progestogen combination treatment		No significant effect compared to placebo (82), may reduce severity of OA (83)	Mixed results (84)
Tibolone	Selective tissue estrogenic activity (85)	Decreases bone loss (86, 87)	Associated with greater risk for stroke (87, 88)
SERM	Selective tissue estrogenic activity without stimulating breast or endothelium tissue (85, 89)	Decreases bone loss maintains bone mineral density (Bazedoxifene, Raloxifene) (86, 87)	May increase risk of some CVDs (Raloxifene, Lasofoxifene, Basedoxifene) (90), sparse evidence suggesting neutral or beneficial effects (Raloxifene) (91)

*CEE, conjugated equine estrogen*.

*SERM, selective tissue estrogen modulator*.

* *Estrogen – for the purposes of this paper, estrogen encompasses references to b-estradiol and 17b-estradiol*.

** *Progesterone – for the purposes of this paper, progesterone encompasses references to progestin (although literature suggests there may be differences in their role, but not enough is known about their risks for OA and CVD specifically)*.

Future clinical directions in examining the role of HT on OA and CVD risk point to the benefits of an individualized approach to prescription, considering the age and time since menopause along with other risk factors (87). Additionally, examining differences across hormone sources, along with other hormone indicators, such as follicle-stimulating hormone and androgens, is warranted (63). Specifically in OA pathology, isolating the effects of sex hormones, such as through examining the effects of low estrogen in pre-menopausal women on OA risk, will clarify the role of sex hormones on maintaining joint health. Finally, given the renewed knowledge of the benefits associated with HT, especially when used early in the post-menopausal period (112), researchers have recommended the continued use of HT for joint and cardioprotective benefits; however, further research is needed on the duration of use and when to cease treatment in post-menopausal women using HT (116).

In addition to an incomplete understanding of the effects of HT with preclinical or existing disease conditions, mixed findings in the literature and inappropriate interpretations of these findings add confusion to the decisions made by women and clinicians (117). When considering the Women's Health Initiative results, initially published in 2002, there have been many misinterpretations of results that have led to significant declines in the prescription and use of HT to treat bothersome menopausal symptoms (117, 118). Future research should be done to further understand HT risks and benefits which would help clarify the information health care teams use to make recommendations and decisions regarding menopausal management.

## Conclusion and Future Directions

OA and CVD collectively contribute to a considerable portion of morbidity and mortality worldwide. The risk for both conditions increase in women following menopause – an inevitable consequence of aging that affects nearly half the world's population for approximately one third of their lives. HT is a method by which women can manage menopausal symptoms during and after this transition; however, there is insufficient understanding of the precise effects that different forms of HT have on a woman's physiology and particularly in the context of OA and CVD risk. As discussed in this review, there exist mixed research findings surrounding effects of HT use on OA and CVD independently. The prevalent comorbidity of OA with CVD has been demonstrated through many studies linking OA and CVD risk factors and pathology, but there has been no research directly examining the intersection of these conditions with HT use. The research findings overlaps and gaps prompt future studies to investigate the relationships between HT with OA and CVD risk in the aging population of post-menopausal women.

Specifically, future research should examine dose, type, and duration of use of HT among women suffering from OA and CVD to help address differential findings in the varying methodologies of previous studies. This approach is of importance, as the earlier treatment and management of OA and CVD risk has the potential to offset morbidity in aging women. Longitudinal studies investigating these factors holistically will provide researchers and clinicians with a better understanding of the role and influence of HT on OA and CVD independently and together, as these different diseases may have different short- and long-term outcomes when considering HT administration. Studies should also focus on women who have undergone hysterectomy and/or ovariectomy procedures, as these are one of the most performed operations in women (119, 120), and the long-term interactions with HT in women who have undergone these procedures are still unclear. Finally, studies must address the influence of sociocultural factors on the pathophysiology and management of menopause and the influence of HT on OA and CVD. The limited research demonstrates significant influences of non-biological considerations that affect the health and quality of care that post-menopausal women receive. Through a comprehensive investigation of existing literature and development of novel research questions, healthcare can provide adequate individualized care. Specifically, the interaction of advanced age and female sex with OA and CVD in conjunction with the roles of HT and menopause in disease progressions prompts future questions to address these gaps in current understanding for health of older women at risk of OA and CVD.

## Author Contributions

YM, JW, and EW were responsible for drafting the article. All authors contributed to editing of the manuscript and contributed to the conception of the project, critical revision of the manuscript, and approving the final article for submission. All authors contributed to the article and approved the submitted version.

## Funding

YM was supported by the McMaster Institute for Research on Aging (MIRA), JW was supported by NSERC CGS-D, EW was supported by MIRA, Canadian Frailty Network, and NSERC CGS-M. AS has received a Grant from Pfizer and has been on the advisory board for Pfizer and Bio-syent. BA-K has Natural Sciences and Engineering Research Council (NSERC) Discovery Grant Award funding (RGPIN-2020-07208). The funding sources were not involved in the study design, writing, reviewing, or decision to submit the manuscript for publication.

## Conflict of Interest

The authors declare that the research was conducted in the absence of any commercial or financial relationships that could be construed as a potential conflict of interest.

## Publisher's Note

All claims expressed in this article are solely those of the authors and do not necessarily represent those of their affiliated organizations, or those of the publisher, the editors and the reviewers. Any product that may be evaluated in this article, or claim that may be made by its manufacturer, is not guaranteed or endorsed by the publisher.
